# Vagus Nerve Suppression in Ischemic Stroke by Carotid Artery Occlusion: Implications for Metabolic Regulation, Cognitive Function, and Gut Microbiome in a Gerbil Model

**DOI:** 10.3390/ijms25147831

**Published:** 2024-07-17

**Authors:** Ting Zhang, Yu Yue, Chen Li, Xuangao Wu, Sunmin Park

**Affiliations:** 1 Korea Department of Bioconvergence, Hoseo University, Asan 31499, Republic of Korea; zhangting92925@gmail.com (T.Z.); yuyue6491@gmail.com (Y.Y.); lic77732@gmail.com (C.L.); niyani0@naver.com (X.W.); 2 Department of Food and Nutrition, Obesity/Diabetes Research Center, Hoseo University, Asan 31499, Republic of Korea

**Keywords:** stroke, gut microbiota, vagus nervous system, memory deficit, insulin

## Abstract

The vagus nerve regulates metabolic homeostasis and mediates gut–brain communication. We hypothesized that vagus nerve dysfunction, induced by truncated vagotomy (VGX) or carotid artery occlusion (AO), would disrupt gut–brain communication and exacerbate metabolic dysregulation, neuroinflammation, and cognitive impairment. This study aimed to test the hypothesis in gerbils fed a high-fat diet. The gerbils were divided into four groups: AO with VGX (AO_VGX), AO without VGX (AO_NVGX), no AO with VGX (NAO_VGX), and no AO without VGX (NAO_NVGX). After 5 weeks on a high-fat diet, the neuronal cell death, neurological severity, hippocampal lipids and inflammation, energy/glucose metabolism, intestinal morphology, and fecal microbiome composition were assessed. AO and VGX increased the neuronal cell death and neurological severity scores associated with increased hippocampal lipid profiles and lipid peroxidation, as well as changes in the inflammatory cytokine expression and brain-derived neurotrophic factor (BDNF) levels. AO and VGX also increased the body weight, visceral fat mass, and insulin resistance and decreased the skeletal muscle mass. The intestinal morphology and microbiome composition were altered, with an increase in the abundance of *Bifidobacterium* and a decrease in *Akkermansia* and *Ruminococcus*. Microbial metagenome functions were also impacted, including glutamatergic synaptic activity, glycogen synthesis, and amino acid biosynthesis. Interestingly, the effects of VGX were not significantly additive with AO, suggesting that AO inhibited the vagus nerve activity, partly offsetting the effects of VGX. In conclusion, AO and VGX exacerbated the dysregulation of energy, glucose, and lipid metabolism, neuroinflammation, and memory deficits, potentially through the modulation of the gut–brain axis. Targeting the gut–brain axis by inhibiting vagus nerve suppression represents a potential therapeutic strategy for ischemic stroke.

## 1. Introduction

Stroke is a leading cause of long-term disability and mortality worldwide. It is characterized by the sudden interruption of blood flow to the brain, resulting in neuronal damage and neurological deficits [1]. Among the various neural pathways implicated in stroke, the vagus nerve, a major component of the parasympathetic nervous system, has garnered attention for its potential role in modulating neurological and metabolic responses to ischemic insults [2]. Vagus nerve stimulation can enhance the functional recovery in individuals who have experienced a stroke [3]. Previous studies have highlighted the intricate relationship between the vagal nerve function, metabolic regulation, cognitive processes, and neurological outcomes following cerebrovascular events [3,4].

Emerging evidence suggests that the disruption of vagal signaling, including vagotomy, can have far-reaching consequences for various physiological systems [4]. Vagotomy has been associated with alterations in energy homeostasis, glucose, lipid metabolism, cognitive function, and memory impairment [5]. These metabolic and neurological disturbances may have significant implications for the development and progression of ischemic stroke. Conversely, vagus nerve stimulation has been shown to improve metabolic processes and ameliorate stroke-related complications, potentially improving rehabilitation outcomes [6]. Interventions that modulate the vagus nerve activity are a novel strategy for managing ischemic stroke and associated comorbidities.

The gut–brain axis, mediated by the vagus nerve, has been recognized as a crucial pathway through which the gut microbiome can influence brain function and behavior. The disruption of this bidirectional communication network, as seen in vagotomy, may contribute to the dysregulation of metabolic processes and cognitive impairment. Furthermore, studies have demonstrated that vagal dysfunction can exacerbate ischemic brain injury and worsen stroke-related outcomes, while stroke-induced brain damage can, in turn, impact vagal tone and function.

Mongolian gerbils are a well-established animal model for studying neurological diseases, including ischemic stroke and epilepsy. Focal cerebral ischemia is induced in the gerbil model through a 5–8 min occlusion of two carotid arteries [7]. Additionally, vagotomy effectively disrupts the vagal communication between the brain and the visceral organs, including the gastrointestinal tract [8]. This allows researchers to examine the potential effects of vagal denervation on energy homeostasis, glucose and lipid metabolism, and cognitive function, including memory impairment [9].

We hypothesized that vagus nerve dysfunction, induced by either vagotomy (VGX) or carotid artery occlusion (AO), would disrupt gut–brain communication and exacerbate metabolic dysregulation, neuroinflammation, and cognitive impairment. We aimed to examine the hypothesis in high-fat fed gerbils. We sought to provide insights that might guide the development of novel therapeutic strategies targeting the vagus nerve for managing stroke and associated comorbidities.

## 2. Results

### 2.1. Brain Cell Death and Clinical Outcomes

**Figure 1:**
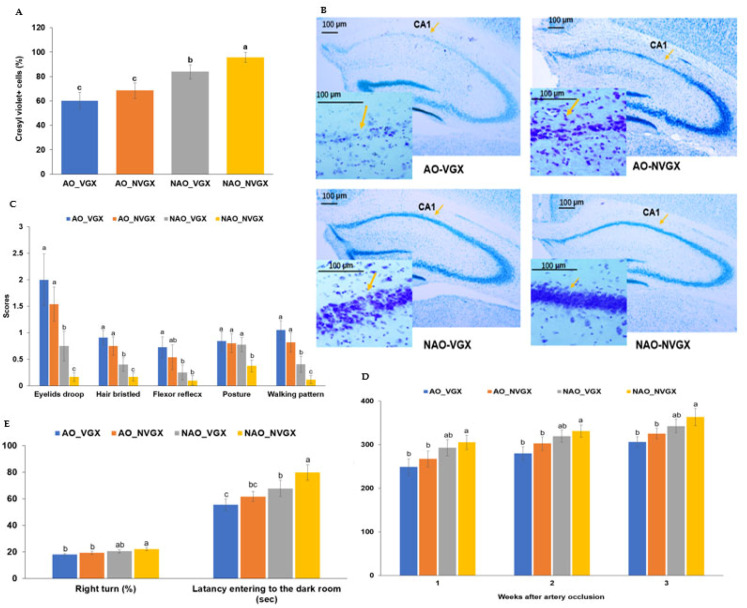
Neuronal live cells and neurological symptoms. (**A**) Neuronal live cells (cresyl violet + cells) in the hippocampus in the 3rd week after carotid artery occlusion (×50 and ×400 magnification; scale bar: 100 μm). (**B**) Images of the neuronal cells in the hippocampus stained by crezyl violet. Yellow arrows indicated live neuronal cells. (**C**) Neurological symptoms in the third week after carotid artery occlusion. (**D**) Grip force in the 1st, 2nd, and 3rd weeks after carotid artery occlusion. (**E**) Y-maze and passive-avoidance tests in the second and third weeks after carotid artery occlusion. a–c: Different superscript letters indicate significant differences between groups by Tukey test at *p* < 0.05.

Gerbils that underwent occlusion of both carotid arteries were designated as AO, while those receiving a sham operation for AO were designated as NAO. Gerbils that underwent VGX were assigned to the VGX, whereas those receiving a sham VGX procedure were assigned to the NVGX. Cresyl violet-positive staining was observed in 64.7, 67.5, 83.5, and 94% of the brain cells in the CA1 regions of the hippocampus in the AO_VGX, AO_NVGX, NAO_VGX, and NAO_NVGX groups, respectively (Figure 1A). While cresyl violet stains various cell types, the majority of these stained cells exhibited morphological characteristics consistent with those of neuronal cells. Carotid artery occlusion significantly decreased the number of live neuronal cells compared to the absence of occlusion. Vagotomy increased neuronal cell death only in the absence of artery occlusion (Figure 1A).

The neurological severity, including droopy eyelids, bristling hair, the flexor reflex, abnormal posture, and walking patterns, was higher in the AO (AO_VGX and AO_NVGX) groups in the second week and decreased in the third week (Supplementary Figure S1). Vagotomy also induced neurological symptoms similar to those observed with AO, as seen in the NAO_VGX group. However, the effects of VGX were less severe in the group with AO (AO_VGX) (Supplementary Figure S1).

The droopy-eyelid, bristling-hair, flexor reflex, and walking-pattern symptom scores were significantly higher in the AO_VGX and AO_NVGX groups than in the NAO_VGX and NAO_NVGX groups in the third week (Figure 1B), suggesting the effects of AO on the neurological symptoms. The NAO_VGX group had weak neurological symptoms, while the NAO_NVGX group did not show the symptoms in the third week (Figure 1B). However, the scores for abnormal posture were significantly lower in the NAO_NVGX group than in the other groups (Figure 1B). The grip force increased in the period after the artery occlusion. It was lower in the AO groups than in the non-AO groups, but the effect of VGX was less severe than that of AO (Figure 1C).

In the evaluation of the memory function using the Y-maze test, the right-turn percentage decreased in the descending order of the NAO_NVGX, NAO_VGX, AO_NVGX, and AO_VGX groups (Figure 1D). The latency entering the dark room was shorter in the groups with artery occlusion than in those without artery occlusion, and it was higher in the NAO_NVGX group than in the NAO_VGX group (Figure 1D). These results indicated that the artery occlusion and vagotomy affected the memory dysfunction, and that the effect of VGX decreased in the gerbils with AO.

### 2.2. Levels of Lipid and Inflammatory Cytokines in the Hippocampus and Liver

The triglyceride and cholesterol levels in the hippocampus increased in the ascending order of the NAO_NVGX, AO_NVGX, AO_VGX, and NAO_VGX groups (Table 1). The glycogen levels in the hippocampus showed an opposite tendency to that of the cholesterol content. The hippocampal lipid peroxide content was also higher in the groups with AO and VGX, and it increased in the ascending order of the NAO_NVGX, NAO_VGX, AO_NVGX, and AO_VGX groups (Table 1). The hippocampal mRNA TNF-α and IL-1β expressions were higher in the groups with AO and VGX. The hippocampal BDNF content was higher in the NAO_VGX, AO_NVGX, and the AO_VGX groups than in the AO_VGX group. Furthermore, the hippocampal HGF and c-met mRNA expressions, which activate neural cell proliferation when an injury occurs, were elevated with AO and VGX. AO increased their expressions more than VGX (Table 1). AO exacerbated hippocampal cell death compared to VGX, and their activation did not recover the neural cell death as much as the NAO_VGX group. The hippocampal acetylcholinesterase activity was higher in the AO_VGX, AO_NVGX, and NAO_VGX groups than in the NAO_NVGX group, and it was highest in the AO_VGX group (Table 1).

The hepatic triglyceride content increased in the ascending order of the AO_NVGX = AO_VGX, NAO_VGX, and NAO_NVGX groups. However, the hepatic cholesterol content was not significantly different among the groups (Table 1). The hepatic glycogen content decreased in the descending order of the NAO_NVGX, AO_NVGX, NAO_VGX, and AO_VGX groups. Therefore, AO and VGX influenced the hepatic lipid and glucose metabolism (Table 1).

The serum TNF-α and IL-1β concentrations were higher in the groups with AO and VGX, and the impact of VGX was diminished in the gerbils with AO. They were the lowest in the NAO_NVGX group (Table 1).

### 2.3. Energy Metabolism

The body weights in the sixth week and the weight gain during the experimental periods were higher in the groups with AO and VGX and were the highest in the AO_VGX group (Table 2). However, the food intake was higher in the groups with VGX but not AO. Thus, AO might decrease energy expenditure. The visceral fat mass was calculated by dividing the epididymal fat and retroperitoneal fat mass by body weight, which was higher in the groups with VGX but not AO. The leg skeletal muscle mass, calculated by dividing the soleus, quadricep, and gastrocnemius mass by body weight, showed a trend opposite to that of the visceral fat mass (Table 2).

### 2.4. Glucose Metabolism

There was no significant variation in the fasting serum glucose concentrations among the groups (Table 2). However, the serum insulin concentrations in the fasting state were higher in the groups with AO and were the lowest in the NAO_NVGX group. The homeostatic model assessment of insulin resistance (HOMA-IR), an insulin resistance index, was lower in the NAO_NVGX group than in the other groups (Table 2).

**Figure 2:**
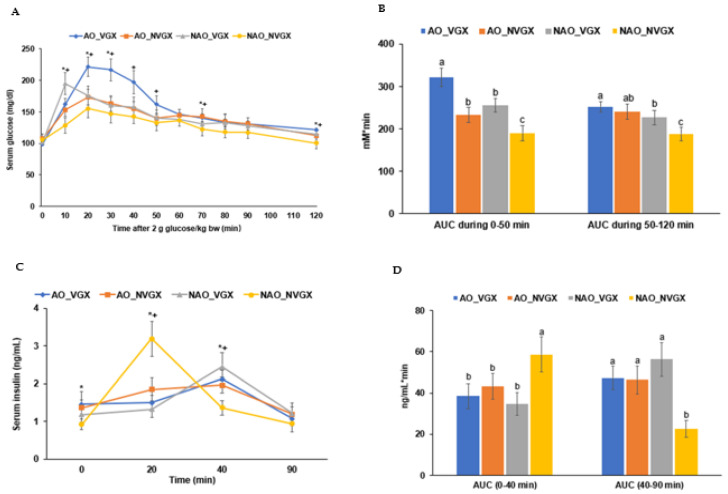
Serum glucose and insulin concentrations during the oral glucose tolerance test were administered orally at 2 g glucose per kg body weight. (**A**) Serum glucose concentrations every 10 min until 90 min and 120 min. (**B**) Areas under the curve (AUCs) of serum glucose concentrations at 0–50 min and 50–120 min. (**C**) Serum insulin concentrations at 0, 20, 40, and 90 min. (**D**) AUCs of serum insulin concentrations at 0–40 min and 40–90 min. * Significant difference by carotid artery occlusion (AO) in two-way ANOVA at *p* < 0.05. + Significant difference by vagotomy (VGX) in two-way ANOVA at *p* < 0.05. a–c: Different superscript letters indicate significant differences between groups by Tukey test at *p* < 0.05.

In the OGTT, after 2 g of glucose per body weight (kg), the serum glucose concentration increased until 20 min. After that point, it decreased rapidly until 50 min. It gradually decreased after that (Figure 2A). The serum glucose levels during the OGTT in the first and second parts of the area under the curve (AUC) were higher in the groups with AO and VGX, and they were the highest in the AO_VGX group and the lowest in the NAO_NVGX group. The AUC of the serum glucose was linked to insulin secretion and sensitivity (Figure 2B).

The serum insulin concentrations increased with the NVGX groups but not the AO groups, and they were the lowest in the AO_NVGX group (Figure 2C). The serum insulin concentrations were the highest at 20 min in the NAO_NVGX group and the highest at 30 min in the other groups. After reaching the peak levels, the serum insulin concentrations decreased (Figure 2C). The AUC of the first part of the serum insulin concentration was much higher in the NVGX group than in the VGX group, regardless of AO. However, the second part showed a trend opposite to that of the first part (Figure 2D). The AUC of the serum insulin in the second part was the lowest in the NAO_NVGX group (Figure 2D). The total AUCs of the serum insulin concentrations were similar in all groups. These results suggest that AO and VGX impaired the first part of insulin secretion, delaying insulin secretion and affecting glucose tolerance.

**Figure 3:**
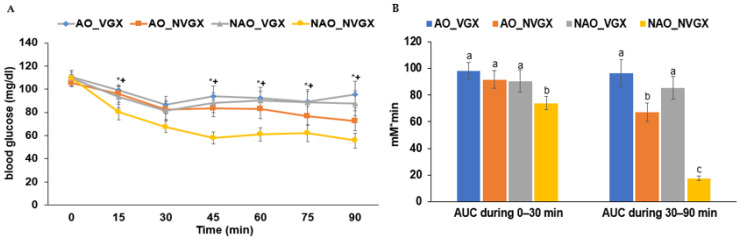
Serum glucose concentrations during an intraperitoneal insulin tolerance test by insulin injected at 1 U per kg body weight. (**A**) Serum glucose concentrations every 15 min until 90 min. (**B**) Areas under the curve (AUCs) of serum glucose concentrations at 0–30 min and 30–90 min. * Significant carotid artery occlusion (AO) difference in two-way ANOVA at *p* < 0.05. ^+^ Significant difference by vagotomy (VGX) in two-way ANOVA at *p* < 0.05. a–c: Different superscript letters indicate significant differences between groups by Tukey test at *p* < 0.05.

At 6 h of fasting, the serum glucose concentrations were not significantly different among the groups. After the intraperitoneal injection of 1 mU of insulin per body weight (kg), the serum glucose declined in all the groups (Figure 3A). The serum glucose concentrations declined in all the groups except in the NAO_NVGX group until 30 min. After 30 or 45 min, the serum glucose concentrations did not change, but they decreased in the AO_NVGX group at 60–90 min. The AUCs of the first phase were significantly lower in the NAO_NVGX group than in the other groups, which were not significantly different (Figure 3B).

### 2.5. Intestinal Morphology

**Figure 4:**
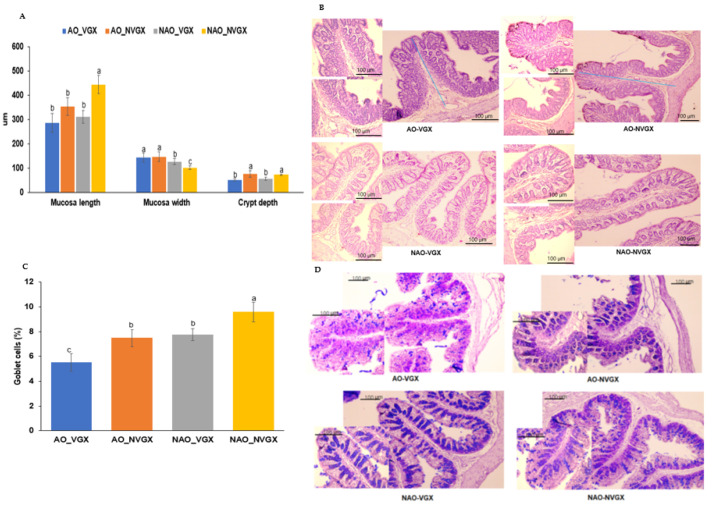
Intestinal morphometry. (**A**) Mucosa length, mucosa width, and crypt depth in the large intestines (**B**) Images of the large intestines stained by H-E (×100 and ×200 magnification; scale bar: 100 μm). The blue line indicates the mucosa length we measured. (**C**) Mucin contents in the large intestines (**D**) Images of the large intestines stained by AB-PAS E (×100 and ×200 magnification; scale bar: 100 μm). a–c: Different superscript letters indicate significant differences between groups by Tukey test at *p* < 0.05.

AO and VGX decreased the mucosa length from the muscularis to the tip of the mucosa in the proximal colon, which was the longest in the NAO_NVGX group. Conversely, the crypt width was higher in the AO-VGX group, and it was the lowest in the NAO_NVGX group (Figure 4A). The crypt height showed a pattern similar to that of the mucosa length (Figure 4A). The number of goblet cells producing mucin was lower in the AO-VGX group, and it was the highest in the NAO_NVGX group (Figure 4B).

### 2.6. Fecal Bacteria

**Figure 5:**
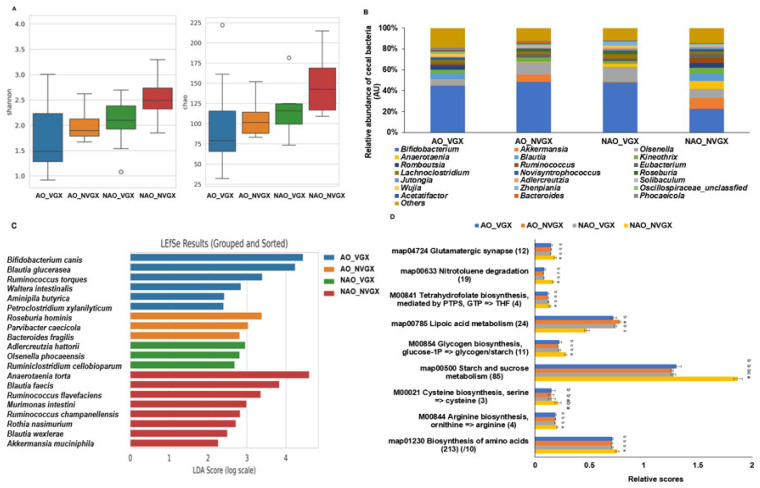
Fecal composition and metagenome function. (**A**) Shannon and Chao indexes. (**B**) Fecal composition at the genus level. (**C**) Primary bacteria at the species level by linear discriminant analysis effect size (LEfSe) analysis. (**D**) Metagenome function by Picrust 2. a–c: Different superscript letters indicate significant differences between groups by Tukey test at *p* < 0.05.

The Shannon and Chao indexes, indicating the alpha-diversity, were higher in the NAO_NVGX group than in the other groups (Figure 5A). Interestingly, at the genus level, the abundance of *Bifidobacterium* was much higher in the AO_VGX, AO_NVGX, and NAO_VGX groups than in the NAO_NVGX group (Figure 5B). However, the abundance of *Akkermansia* was higher in the AO_NVGX and NAO_NVGX groups than in the other groups. The abundances of *Anaerotaenia* and *Ruminoccocus* were higher in the NAO_NVGX group than in the other groups. The primary bacteria in each group were selected in the linear discriminant analysis effect size (LEfSe) analysis at the species level. The AO_VGX group showed abundances of *Bifidobacterium canis*, *Blautia glucerasea*, *Ruminococcus torques*, *Waltera intestinalis*, *Aminipila butyrica*, and *Petroclostridium xylanilyticum* (Figure 5C). The AO_NVGX group contained *Roseburia hominis*, *Parvibactere caecicola*, and *Bacteroides fragilis* in abundance. The NAO_VGX group included *Adlercreutzia hattorii*, *Olsenella phocaeensis*, and *Ruminiclostridium cellobioparum*. The NAO_NVGX group contained *Anaerotaenia torta*, *Blautia faecis*, *Ruminococcus flavefaciens*, *Murimonas intestine*, *Ruminococcus champanellensis*, *Rothia nasimurium*, *Blautia wexlerae*, and *Akkermansia muciniphila* (Figure 5C).

In the metagenome analysis, the glutamatergic synapse and nitrotoluene showed higher scores in the NAO_NVGX group than in the other groups (Figure 5D). However, the lipoic acid metabolism was lower in the NAO_NVGX group than in the other groups, and the nitrotoluene degradation showed a trend opposite to that of the lipoic acid metabolism. The starch and sucrose metabolism and glycogen biosynthesis were much higher in the NAO_NVGX group than in the other groups (Figure 5D). The amino acid biosynthesis was also higher in the NAO_NVGX group than in the other groups. Among these, the cysteine and arginine biosynthesis and tetrahydrofolate biosynthesis were higher in the NAO_NVGX group than in the other groups (Figure 5D).

## 3. Discussion

This study found that both AO and VGX decreased the neuronal viability and neurological severity scores, though the effects were not additively worsened. These neurological changes were associated with altered hippocampal lipid profiles, including increased triglycerides and cholesterol and changes in the hippocampal glycogen content, lipid peroxidation, inflammatory cytokine expression, and neurotrophic factor BDNF. Additionally, AO and VGX influenced the whole-body metabolism, leading to an increase in the body weight and visceral fat, a decrease in the skeletal muscle mass, and an elevation in the serum insulin concentration and insulin resistance. Furthermore, AO and VGX modified the morphology of the large intestines and altered the diversity and composition of their fecal microbiomes with changes in the microbial metabolic pathways.

The vagus nerve is a primary component of the parasympathetic nervous system, which plays a crucial role in regulating energy balance and glucose and lipid metabolism [10]. Furthermore, carotid artery occlusion suppresses vagus nerve activation in the parasympathetic nervous system [11], and vagus nerve stimulation attenuates cerebral ischemia symptoms [12]. This study showed that VGX increased the body weight with elevated food intake, and it additively worsened the body weight and fat mass in the gerbils. However, AO by itself did not increase the body weight and food intake. This suggests that AO by itself partially suppressed the vagus nerve activation. VGX stimulates food intake and increases body weight by suppressing vagus nerve activity and modulating appetite-regulating hormones and neuropeptides, such as leptin, ghrelin, glucagon-like peptide-1, and neuropeptide Y, as reported in previous studies [10,13,14]. However, the relationship between VGX and energy metabolism remains controversial, and it may be linked to vagotomy [14,15].

In addition to body weight, the vagus nerve is involved in body composition, including the body fat and skeletal muscle contents [10,16]. Vagus nerve stimulation reduces food intake, body weight gain, and mesenteric adipose tissue in rats, and the reduction in adipose tissue by visceral rat adipocytes is greater than the gain in body weight [10]. These actions of the vagus nerve are related to peroxisome proliferator-activated receptor-α activation. The present study showed that VGX elevated the visceral adipose tissue and decreased the skeletal muscle mass in the legs of gerbils. AO, in contrast, did not influence these parameters. However, AO exacerbated the elevated the visceral fat mass in the VGX gerbils (the AO_VGX group). Therefore, the vagus nerve may be pivotal in modulating body composition. Previous studies have demonstrated that stroke is linked to sarcopenia and low muscle mass, and sarcopenia is frequently observed in stroke survivors and occurs in the early phase, post-stroke [17,18].

AO and VGX are known to be related to the regulation of glucose and lipid metabolism through their effects on the liver, pancreas, and other organs. The present study showed that AO and VGX impaired glucose tolerance with lower insulin secretion and higher insulin resistance, observed in the OGTT and ITT. The first and second parts of the serum glucose concentration were much higher in the AO_VGX group than in the NAO_NVGX group. Among the groups, the peak value of the serum glucose concentration was the highest in the AO_VGX group. The results of the serum insulin concentrations were the opposite to those seen with the glucose concentrations. The NAO_NVGX group showed a marked increase in its serum insulin concentrations at 20 min and a decrease from 40–90 min, but the other groups showed lower peaks of serum insulin concentrations, and the peak was at 40 min. VGX led to impaired glucose tolerance and a significant increase in glucose production due to increased glycogenolysis and triglyceride and cholesterol deposition by the liver. The present study suggests that VGX and AO synergistically impaired insulin secretion and increased insulin resistance. Consistent with the present study, previous studies have shown that AO causes a disturbance in glucose metabolism by decreasing insulin secretion, resulting in hyperglycemia post-stroke, thereby exacerbating stroke outcomes [19]. VGX can reduce insulin secretion from the pancreas due to the absence of a signal from the vagus nerve [20]. Furthermore, the loss of vagal stimulation can decrease glucose uptake by the liver and other tissues, further contributing to elevated blood glucose levels. VGX also increases gluconeogenesis in the liver. In short, VGX impairs glucose and lipid metabolism.

AO leading to cerebral ischemia and hypoxia induces neuronal cell death, especially in the hippocampus [21]. VGX can also impair memory function through various mechanisms, such as by disrupting the cholinergic system, hippocampal function, neurogenesis, neuroplasticity, and inflammatory processes [22]. It is also involved in the microbiota–hippocampus pathway through the vagus nerve, known as the gut–brain axis [23]. The present study showed results consistent with those of earlier studies that found that AO and VGX elevated neuronal cell death, memory deficits, and neurological outcomes. However, no previous study has investigated the interaction between AO and VGX. We note that we measured neuronal cell death using the cresyl violet-staining method. While this method allows for the visualization of the brain cell architecture, it does not explicitly identify neuronal subtypes. Our interpretations are based on the morphological characteristics typical of neurons but that may include other cell types. This limitation should be considered when interpreting our results. Future studies employing more specific neuronal markers and direct measurements of the vagal activity in AO models could help clarify these mechanisms.

AO leads to the inducement of neurological symptoms and memory deficits. However, our study demonstrated that VGX did not significantly exacerbate the neuronal cell death, neurological outcomes, memory deficits, or grip force reductions in the AO model, despite the initial expectation of an additive or synergistic effect. This lack of significant exacerbation might be related to the suppressed vagus nerve activity caused by AO itself. AO may lead to the dysfunction of the autonomic nervous system, including reduced vagal tone, which potentially masks the effects of surgical vagotomy. Previous studies have demonstrated that vagus nerve stimulation significantly reduces the extent of stroke-induced lesions in brain parenchyma [24]. Furthermore, vagus nerve stimulation reduces the stroke volume, decreases inflammation, and attenuates neurological deficits in ischemic stroke models through the cholinergic pathway [25,26]. Therefore, the absence of significant exacerbation of AO-induced neurological symptoms by VGX might be linked to the decreased vagus nerve activity caused by AO.

The vagus nerve plays a crucial role in modulating gut microbiota through the gut–brain axis, which involves bidirectional communication between the gastrointestinal tract and the central nervous system. Disruption of this axis due to conditions like AO and VGX can potentially impact the gut microbiota composition. Additionally, ischemic brain injury and an impairment of the vagus nerve can disrupt the autonomic nervous system, which regulates gut motility and secretions, indirectly affecting the gut microbiota. Ischemic stroke increases the intestinal mucosal permeability and the mucosal layer in the intestinal mucosal epithelium [27,28,29]. Consistent with previous studies [27,28,29], the present study showed that AO and VGX decreased the intestinal mucosa length and mucin content while increasing the crypt width, indicating an unfavorable gut environment. However, no significant additive changes in the intestinal morphology were observed, suggesting that AO and VGX may act through similar mechanisms. AO might develop an unfavorable gut environment, which might be linked to the vagus nerve through disturbances in the gut–brain axis.

This study also demonstrates that elevated mRNA expressions of *HGF* and its receptor *c-met* in the AO and VGX groups indicated neuroprotective and regenerative responses to neuronal injury, with AO showing a more potent effect. The HGF/c-met pathway is crucial for the tissue architecture during embryonic development and adult tissue homeostasis, aiding in repairing injured organs and blood vessels via mitogenic, angiogenic, anti-apoptotic, and anti-inflammatory signals [30]. The c-met receptor enhances neurite outgrowth in rat hippocampal neurons, underscoring HGF’s role in neuron maturation and function [31,32]. While HGF production is critical for hippocampal restoration, its secretion can be transient or insufficient, explaining why HGF supplementation aids in tissue regeneration, including neurons [33]. The study also found elevated hippocampal mRNA expressions of *TNF-α* and *IL-1β* in the AO and VGX groups, both positive regulators of HGF. HGF’s neurotrophic function, acting against cerebral ischemia in rats, reduces the infarct volume and neuronal death following AO [34]. AO and VGX impaired glucose tolerance, with lower insulin secretion and higher insulin resistance. HGF is pivotal in compensatory mechanisms for insulin resistance, correlating with β-cell mass increases and improved insulin signaling [35]. Increased mRNA expressions of *HGF* and *c-met* may reduce hippocampal neuronal cell death, offering potential therapeutic benefits for cerebral ischemia. Therefore, AO appears more effective than VGX in upregulating *HGF* and *c-met*, suggesting the potential of targeting the HGF/c-met pathway for neuroprotection and metabolic regulation in ischemic conditions.

Interestingly, the changes in the gut microbiota composition mirrored the alterations in the intestinal morphology. The α-diversity indices (Shannon and Chao) were higher in the NAO_NVGX group compared to the others, indicating a more diverse gut microbiota in the absence of AO and VGX. Notably, the abundance of *Bifidobacterium* was significantly higher in the AO_VGX, AO_NVGX, and NAO_VGX groups than in the NAO_NVGX group. In contrast, the relative abundances of *Akkermansia, Anerofaenia*, and *Ruminococcus* displayed an opposing pattern among the groups to that of *Bifidobacterium*. However, previous studies have demonstrated inconsistent results in gut microbiota modulation in ischemic stroke [36,37]. The enrichment of *Bifidobacterium* in the AO and VGX groups is consistent with some previous reports. However, it is still controversial. This suggests that vagus nerve disturbances, such as those observed in Alzheimer’s disease and carotid artery occlusion, can increase the *Bifidobacterium* levels, potentially due to altered intestinal permeability [7,38]. Treatment with beneficial *Bifidobacterium* species, such as *B. longum* and *B. breve*, has improved ischemic stroke symptoms [39], and it may be linked to a better environment for the settlement of patients. The increases in *Bifidobacterium* may protect against the exacerbation of neurodegenerative diseases, including stroke. Furthermore, the colonization and cell number of the *Bifidobacterium* species in the human intestine vary with age [40]. The results suggest that changes in the abundance of *Bifidobacteria* may modulate vagus nerve activity.

## 4. Materials and Methods

### 4.1. Animals and Diets

Male Mongolian gerbils (*Meriones unguiculatus*), aged seven weeks, were purchased from DaehanBio (Eumsung, Korea) and allowed to acclimate in the animal facility for one week. Throughout the acclimation and experimental period, the gerbils were maintained under controlled environmental conditions of a 23 °C temperature, 60% humidity, and a 12 h light/dark cycle, with access to food and water ad libitum. The animal study was approved by the Institutional Animal Care and Use Committee of Hoseo University (HSIACUC-22-051), and it followed the Guide for the Care and Use of Laboratory Animals (8th edition) issued by the National Institutes of Health.

The gerbils were fed a high-fat diet in entire experimental periods based on the semi-purified AIN-93 diet composition, with detailed nutrient information provided in Supplementary Table S1. The diet consisted of 40, 17, and 43 energy percent (En%) from carbohydrates, protein, and fats, respectively, based on total macronutrient energy. Additionally, non-energy nutrients were included: cholesterol (2.5%), cellulose (3.4%), minerals (3.5%), and vitamins (1.0%), based on total diet weight.

### 4.2. Vagotomy and Transient Forebrain Ischemia

Prior to surgery, the gerbils were fasted overnight. During the vagotomy procedure, the animals received intramuscular anesthesia with a ketamine (Pfizer, New York, NY, USA) and xylazine (BD Bioscience, Franklin Lake, NJ, USA) cocktail (100 mg/kg and 10 mg/kg, respectively). Subsequently, bilateral total subdiaphragmatic vagotomy surgery was performed [15]. After conducting a midline laparotomy to expose the abdominal cavity below the diaphragm, the liver was gently retracted to expose the esophageal hiatus. The anterior and posterior trunk vagus nerves were isolated as they traversed the esophageal hiatus to ligate and fix them, and a 1 cm segment from each trunk was resected to complete the bilateral total subdiaphragmatic vagotomy [15]. This procedure effectively disrupts vagal communication between the brain and visceral organs, including the stomach, small and large intestines, pancreas, and liver. However, vagal innervation to organs such as the oral cavity and pharynx remains intact. In sham surgeries, the trunk vagus nerves were exposed but not ligated, and the sham gerbils had intact vagal communication. Post-surgery, all animals were housed individually in cages.

After 2 weeks post-VGX-surgery, transient ischemic stroke was induced by occluding two common carotid arteries using aneurysm clips for 8 min following a midline incision of the neck skin. During artery occlusion, rectal temperature was monitored and maintained at 37 ± 0.5 °C using a rectal temperature probe (TR-100; Fine Science Tools, Foster City, CA, USA). Post-occlusion, the gerbils were placed in a thermal incubator for 12 h to maintain body temperature [41,42]. Sham-operated animals underwent identical surgical procedures to serve as controls for comparison.

### 4.3. Experimental Design and Metabolic Analysis

Forty gerbils were randomly assigned to one of four groups: (1) AO in both carotid arteries + VGX (AO_VGX); (2) AO + no VGX (AO_NVGX); (3) no AO + VGX (NAO_VGX); and (4) no AO + no VGX (NAO_NVGX). Gerbils in the NAO or NVGX groups underwent sham surgery. All the rats were fed a high-fat diet.

**Figure 6:**
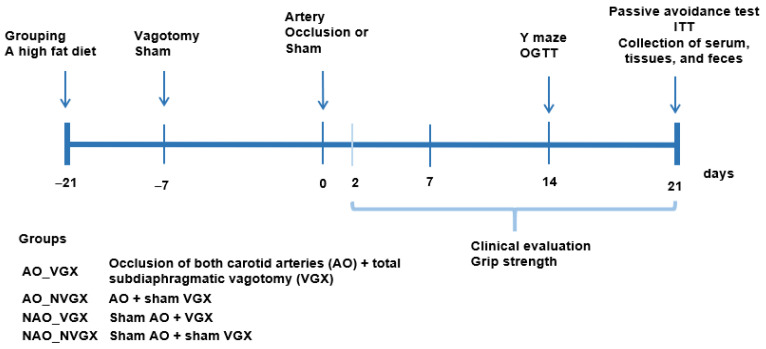
Experimental design.

Figure 6 illustrates the experimental design. Following three weeks of consuming their assigned diets, artery occlusion was performed, and clinical neurological symptoms were assessed at 2, 7, 14, and 21 days post-occlusion. Y-maze testing was conducted at one week, an oral glucose tolerance test (OGTT) at 2 weeks, and an intraperitoneal insulin tolerance test (IPITT) at 3 weeks post-occlusion. During the OGTT, the animals received an oral dose of 2 g glucose/kg body weight following an overnight fast, and blood samples were collected to measure glucose and serum insulin concentrations, as previously described [43]. Following the completion of the OGTT, animals were provided with food. After a six-hour fast, the IPITT was conducted the following day, as previously described [43]. Y-maze and passive-avoidance tests were also performed at 2 and 3 weeks post-occlusion, and grip strength assessments were conducted at 1 week, 2 weeks, and 3 weeks post-occlusion. Weekly measurements of food intake, body weight, and overnight-fasted serum glucose levels were recorded. Serum glucose and insulin levels were determined using a Glucose Analyzer II (Beckman, Palo Alto, CA, USA) and an ultrasensitive rat mouse insulin kit (Crystal Chem, Elk Grove Village, IL, USA), respectively.

The day after the passive-avoidance test on day 21, gerbils were fasted overnight and then euthanized with carbon dioxide asphyxiation. Their organs were dissected, and their weights were recorded. Blood was collected from the vena cava and portal veins, and serum was separated by centrifugation at 500× *g*. The skull was carefully opened, starting from the base (foramen magnum) and extending along the midline towards the nose using fine scissors or a bone saw. The skull cap was gently removed to expose the brain, which was then carefully lifted out using a small spatula or fine forceps. The brains of six randomly selected gerbils from each group were immersed in a 20% sucrose solution at 4 °C overnight and subsequently frozen at −20 °C. The brain organ was positioned ventral-side-up on a chilled dissection tray under a dissecting microscope for the remaining four gerbils per group. With a fine knife, the four-sided parts near the hippocampus area and some left parts, such as the cerebellum and cortex, were gently removed with fine scissors, forceps, and a micro-spatula to provide a clearer view of the hippocampal region. The hippocampus was then dissected and divided into two portions. One portion was lysed with radioimmunoprecipitation assay (RIPA) buffer, and the supernatants were collected to measure the triglyceride, cholesterol, and lipid peroxide levels using spectrophotometric kits (DoGenBio, Ansan, Republic of Korea). The supernatants were digested with α-amyloglucosidase (Sigma Co., St. Loise, MO, USA), and the glucose content was measured to calculate the glycogen levels using a glucose spectrophotometric kit (DoGenBio). Acetylcholinesterase (AChE) activity was assessed using a rodent AChE enzyme-linked immunosorbent assay (ELISA) kit (Elabscience, Huston, TX, USA). Serum glucose concentrations were measured with a Beckman glucose analyzer. Serum levels of tumor necrosis factor-α (TNF-α), interleukin (IL)-1β, and insulin were measured using the respective ELISA kits from Invitrogen (Waltham, MA, USA).

### 4.4. Neurological Severity Score and Grip Strength

Neurological severity was clinically assessed at 1 week, 2 weeks, and 3 weeks post-occlusion using the following criteria: eyelid droop (0, no symptom; 1, partial droop of one eyelid; 2, complete droop of one eyelid; 3, partial droop of both eyelids; 4, complete droop of both eyelids); hair bristling (0, no symptom; 1, bristling hair); flexor reflex (0, no symptom; 1, slight withdrawal of hind limbs when pinched; 2, no withdrawal of hind limbs when pinched); posture (0, normal; 1, hunched); walking pattern (0, normal; 1, slow; 2, no walking). Forelimb grip strength was assessed using a Grip Strength Meter (GPM-100; Melquest, Toyama, Japan) [44]. Gerbils grasped a bar mounted on a force gauge, and a researcher gently pulled the tail. Peak pull force was recorded using a digital force transducer.

### 4.5. Assessment of Memory Impairment Using Passive-Avoidance and Y-Maze Tests

A passive-avoidance test was conducted using a two-compartment dark/light shuttle-box apparatus [45] to evaluate memory impairment. In the first trial, when a gerbil entered the dark chamber, it received an electric shock (75 V, 0.2 mA, 50 Hz) for five seconds. After an 8 h interval, the second trial was conducted under the same conditions. Sixteen hours after the second trial, the latency time to enter the dark chamber was measured, similar to the first trial but without the electric stimulation to the feet [45]. Latency periods to enter the dark room were recorded for up to 600 s.

Short-term memory was assessed using the Y-maze test, which consisted of a horizontal Y-shaped maze with three arms measuring 50.5 cm in length, 20 cm in width, and 20 cm in height [45]. A gerbil was placed in one arm, and its movements in each arm were observed for 8 minutes. The number of correct consecutive entries into each arm of the Y maze was recorded, and the percentage of correct consecutive alternations was calculated. A higher percentage indicated better short-term memory performance.

### 4.6. Organ Collection and Hippocampal mRNA Expression Analysis

The total RNA of the hippocampus from five gerbils was isolated using TRIzol reagent (Life Technologies, Rockville, MD, USA). Subsequently, cDNA was synthesized from 1 μg of total RNA extracted from individual gerbils using a Superscript III reverse transcriptase kit (Life Science Technology). As previously described [45], equal amounts of cDNA and primers for specific genes were combined with the SYBR Green mix (Bio-Rad, Richmond, CA, USA) in duplicate and subjected to amplification using a real-time PCR instrument (Bio-Rad). Primers for genes, including brain-derived neurotrophic factor (BDNF), ciliary neurotrophic factor (CNTF), Tau, tumor necrosis factor (TNF)-α, interleukin (IL)-1β, hepatic growth factor (HGF), and c-met, were utilized as described in prior studies [44]. Each sample’s cycle threshold (CT) was determined, and the gene expression levels in unknown samples were quantified using the comparative CT method (ΔΔCT method). The results are presented as 2^−ΔΔCT^.

### 4.7. Cresyl Violet Staining to Assess Neuronal Live Cells 

Cresyl violet staining was performed on the hippocampal sections of the gerbils to assess the percentage of neuronal live cells. The brains were immersed overnight in a 30% sucrose solution and subsequently frozen. Frozen brains were sectioned serially at 30 µm thickness using a cryostat (Leica, Wetzlar, Germany), and the sections were then mounted on gelatin-coated microscopy slides. After staining with a 0.1% cresyl violet solution (Merck, Darmstadt, Hessen, Germany) in 0.6% glacial acetic acid (Sigma Co.) for 2 min at room temperature, the brain sections were rinsed twice in distilled water. The fixed brain tissues were then dehydrated by immersion in a graded series of ethanol at room temperature and finally mounted with Permount (Fisher Scientific Inc., Pittsburgh, PA, USA).

### 4.8. H-E and PAS Staining of Large Intestines

The proximal colon of each rat was dissected below the cecum. The lumen was gently flushed with PBS to remove fecal matter, followed by a flush with 4% paraformaldehyde. The washed proximal colon was then immersed in 4% paraformaldehyde and fixed overnight at 4 °C [13]. Two serial 5 μm paraffin-embedded proximal colon sections were selected from the seventh or eighth sections to avoid counting the same site twice. The section was stained using hematoxylin–eosin (H-E) and Alcian blue–perchloric acid (PAS) staining. After staining, the mucosa’s length above the muscularis mucosa in the proximal colon and the crypt’s width and height were measured in two H-E-stained sections using a Zeiss Axiovert microscope with the DIXI Imaging solution. Each value was the average of the mucosa length and crypt width and height. The percentage of goblet cells producing mucin, as indicated by blue staining, was counted in the Alcian blue–PAS-stained sections, and its percentage of the intestine area was calculated.

### 4.9. Next-Generation Sequencing (NGS) Analysis of Gut Microbiomes

The fecal composition of the gut microbiome was assessed by an NGS analysis of cecum samples, following established protocols [43]. Mothur v.1.36 software was employed to analyze the 16 S amplicon sequencing [43]. We adhered to the MiSeq system guidelines for bacterial identification and enumeration in all fecal samples. Sequences were aligned using the SILVA reference alignment v.12350, and operational taxonomic units (OTUs) were selected with a 98% identity threshold and taxonomically classified by consensus using the SILVA reference database [43]. Principal coordinate analysis (PCoA) was performed using the R package 4.2.3, with the OTU abundance table converted to relative abundance for analysis.

### 4.10. Statistical Analysis

SAS software version 5 was utilized for statistical analysis. Results are presented as means ± standard deviations (SDs). The normality of the data distribution was assessed using univariate analysis with the Shapiro–Wilk test. Homogeneity of variance was tested using Levene’s test, which checks whether the variances across groups are equal. A *p*-value > 0.05 for both tests indicated that the assumptions were met. All data met the normal-distribution and homogeneity-of-variance assumptions. The significance of the AO and VGX effects on the cerebral ischemia symptoms was assessed using a two-way analysis of variance (ANOVA), where AO and VGX were the two factors analyzed. Tukey’s post hoc test was conducted to identify differences among groups if the ANOVA was significantly different. Differences among groups with a *p*-value < 0.05 were deemed statistically significant.

## 5. Conclusions

AO exacerbated the neuronal cell death, neurological deficits, memory impairment, and metabolic dysregulation in a gerbil model. Remarkably, VGX did not further exacerbate the neurological outcomes in the AO group, suggesting that artery occlusion by itself may have suppressed the vagus nerve activity, providing a vagotomy-like effect. The results indicate that the vagus nerve acts as a pivotal mediator of the symptoms associated with artery occlusion, potentially through the modulation of the gut–brain axis. Additionally, artery occlusion and vagotomy altered the intestinal morphology and gut microbiota composition, with a notable increase in the *Bifidobacterium* abundance. The enrichment of *Bifidobacterium* may be linked to vagus nerve modulation and could protect against neurodegeneration. Furthermore, the colonization and abundance of *Bifidobacterium* species varied with aging, reinforcing the association between the vagus nerve and gut microbiota dynamics. Overall, these findings highlight the complex interplay between the vagus nerve, gut microbiota, and neurological and metabolic outcomes in ischemic stroke, underscoring the potential therapeutic implications of targeting the gut–brain axis.

## Figures and Tables

**Table 1:** Hippocampal lipid and inflammatory indexes.

	AO_VGX	AO_NVGX	NAO_VGX	NAO_NVGX
Hippocampal TG (mg/g)	19.5 ± 1.06 ^a^	17.9 ± 1.18 ^b^	18.3 ± 0.95 ^b^	16.4 ± 1.26 ^c^*^+^
Hippocampal cholesterol (mg/g)	26.8 ± 1.52 ^a^	24.5 ± 1.65 ^b^	25.4 ± 1.64 ^ab^	22.1 ± 1.05 ^c^*^+^
Hippocampal glycogen (mg/g)	18.0 ± 1.95 ^a^	20.6 ± 1.63 ^b^	20.1 ± 1.24 ^b^	26.0 ± 1.41 ^c^*^+^
Hippocampal lipid peroxides (MDA μmol/g tissue)	0.62 ± 0.08 ^a^	0.57 ± 0.07 ^ab^	0.51 ± 0.08 ^b^	0.34 ± 0.06 ^c^*^+^
Hippocampal acetylcholinesterase (U/mg protein)	0.29 ± 0.06 ^a^	0.26 ± 0.05 ^ab^	0.22 ± 0.05 ^b^	0.11 ± 0.04 ^c^*^+^
Hippocampal mRNA TNF- (AU)	2.7 ± 0.7 ^a^	2.2 ± 0.6 ^ab^	1.8 ± 0.6 ^b^	1.0 ± 0.0 ^c^*^+^
Hippocampal mRNA IL-β (AU)	2.2 ± 0.5 ^a^	1.9 ± 0.5 ^ab^	1.5 ± 0.4 ^b^	1.0 ± 0.0 ^c^*^+^
Hippocampal mRNA BDNF (AU)	0.63 ± 0.08 ^c^	0.69 ± 0.09 ^bc^	0.78 ± 0.11 ^b^	1.0 ± 0.0 ^a^*^+^
Hippocampal mRNA HGF (AU)	2.67 ± 0.21 ^a^	2.52 ± 0.23 ^ab^	1.94 ± 0.17 ^b^	1.0 ± 0.0 ^c^*^+^
Hippocampal mRNA c-met (AU)	1.96 ± 0.16 ^a^	1.85 ± 0.14 ^ab^	1.67 ± 0.15 ^b^	1.0 ± 0.0 ^a^
Hepatic TG (mg/g)	43.4 ± 3.64 ^a^	42.2 ± 4.83 ^a^	37.6 ± 2.26 ^b^	32.5 ± 3.27 ^c^*^+^
Hepatic cholesterol (mg/g)	29.0 ± 3.45	30.9 ± 3.73	28.5 ± 2.25	29.4 ± 3.56
Hepatic glycogen (mg/g)	39.3 ± 6.61 ^c^	51.9 ± 8.53 ^b^	47.9 ± 4.85 ^b^	62.5 ± 7.26 ^a^*^+^
Serum TNF-α (pg/dL)	28.5 ± 3.03 ^a^	27.9 ± 2.87 ^a^	24.3 ± 2.51 ^b^	18.7 ± 2.24 ^c^*^+^
Serum IL-1β (pg/dL)	11.8 ± 1.29 ^a^	10.9 ± 1.22 ^ab^	10.1 ± 1.08 ^b^	8.21 ± 0.97 ^c^*^+^

Values represent means ± SDs (*n* = 10). TG, triglyceride; TNF, tumor-necrosis factor; IL, interleukin; MDA, malondialdehyde. * Significant difference by carotid artery occlusion (AO) in two-way ANOVA at *p* < 0.05. ^+^ Significant difference by vagotomy (VGX) in two-way ANOVA at *p* < 0.05. a–c: Different superscript letters indicate significant differences between groups by Tukey test at *p* < 0.05.

**Table 2:** Energy, glucose, and lipid metabolism.

	AO_VGX	AO_NVGX	NAO_VGX	NAO_NVGX
Body weight (g)	64.3 ± 3.92 ^a^	54.3 ± 3.21 ^c^	58.3 ± 3.38 ^b^	52.3 ± 3.87 ^c^*^+^
Body weight gain (g)	14.1 ± 1.38 ^a^	5.82 ± 0.94 ^c^	9.53 ± 1.16 ^b^	4.74 ± 0.72 ^c^*^+^
Food intake (g/day)	5.44 ± 0.43 ^a^	4.34 ± 0.41 ^b^	5.50 ± 0.54 ^a^	4.47 ± 0.57 ^b+^
Visceral fat (% bw)	1.19 ± 0.21 ^a^	0.75 ± 0.26 ^c^	1.01 ± 0.15 ^b^	0.74 ± 0.23 ^c+^
Skeletal muscle (% bw)	2.01 ± 0.12 ^b^	2.38 ± 0.22 ^a^	2.02 ± 0.18 ^b^	2.42 ± 0.16 ^a+^
Serum glucose (mg/dL)	106 ± 2.67	105 ± 4.47	102 ± 5.01	97.9 ± 4.84
Serum insulin (ng/mL)	0.95 ± 0.15 ^a^	0.98 ± 0.16 ^a^	0.96 ± 0.17 ^a^	0.79 ± 0.11 ^b^*
HOMA_IR	6.3 ± 0.77 ^a^	6.49 ± 0.91 ^a^	6.1 ± 0.72 ^a^	5.0 ± 0.59 ^b^*^+^
Serum total cholesterol (mg/dL)	233 ± 24.5 ^a^	196 ± 26.2 ^b^	250 ± 15.3 ^a^	164 ± 26.9 ^c^*^+^
Serum HDL (mg/dL)	36.6 ± 5.1	36.8 ± 4.2	37.4 ± 5.8	38.5 ± 4.9
Serum triglycerides (mg/dL)	109 ± 13.2 ^a^	105 ± 6.8 ^a^	90.3 ± 10.6 ^b^	85.4 ± 9.2 ^b^*^+^

Values represent means ± SDs (*n* = 10). HOMA-IR, homeostatic model assessment for insulin resistance. HDL, high-density lipoprotein. * Significant difference by carotid artery occlusion (AO) in two-way ANOVA at *p* < 0.05. ^+^ Significant difference by vagotomy (VGX) in two-way ANOVA at *p* < 0.05. a–c: Different superscript letters indicate significant differences between groups by Tukey test at *p* < 0.05.

## Data Availability

The data will be available upon the request to the corresponding author.

## Supplementary Materials

The following supporting information can be downloaded at https://www.mdpi.com/article/10.3390/ijms25147831/s1.
