# Supplementary material for: Vagus Nerve Suppression in Ischemic Stroke by Carotid Artery Occlusion: Implications for Metabolic Regulation, Cognitive Function, and Gut Microbiome in a Gerbil Model
Source: Int J Mol Sci. 2024 Jul 17;25(14):7831. doi: 10.3390/ijms25147831 (PMC11276658; doi:10.3390/ijms25147831)

Supplementary Figure S1. Weekly changes of neurological symptoms after artery occlusion

A. Eyelid drooped

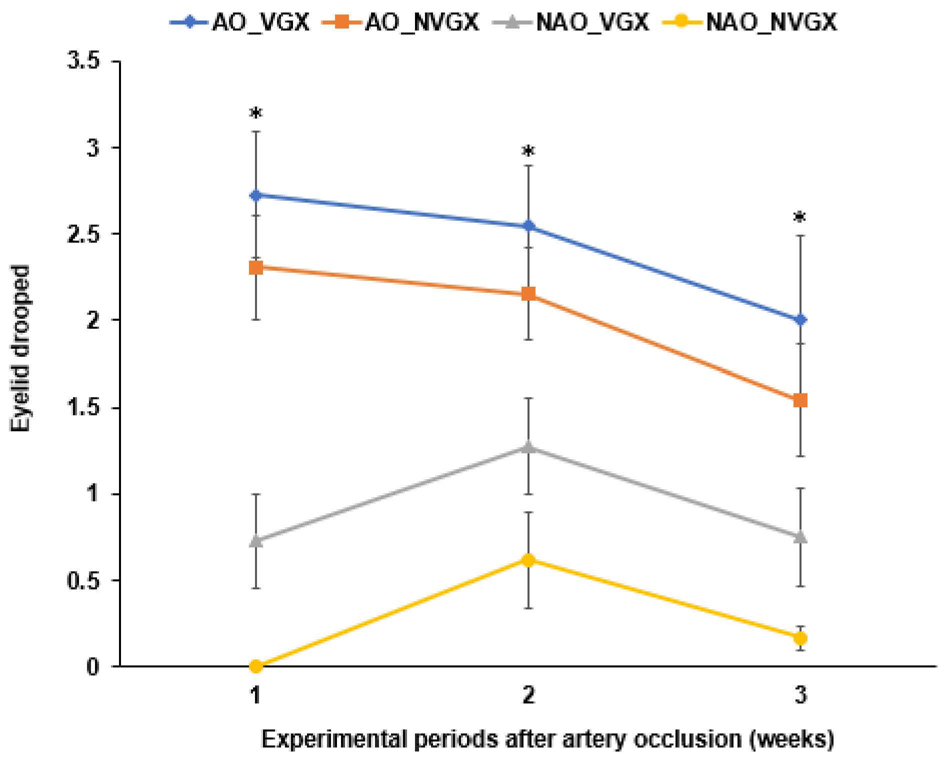

B. Hair bristling

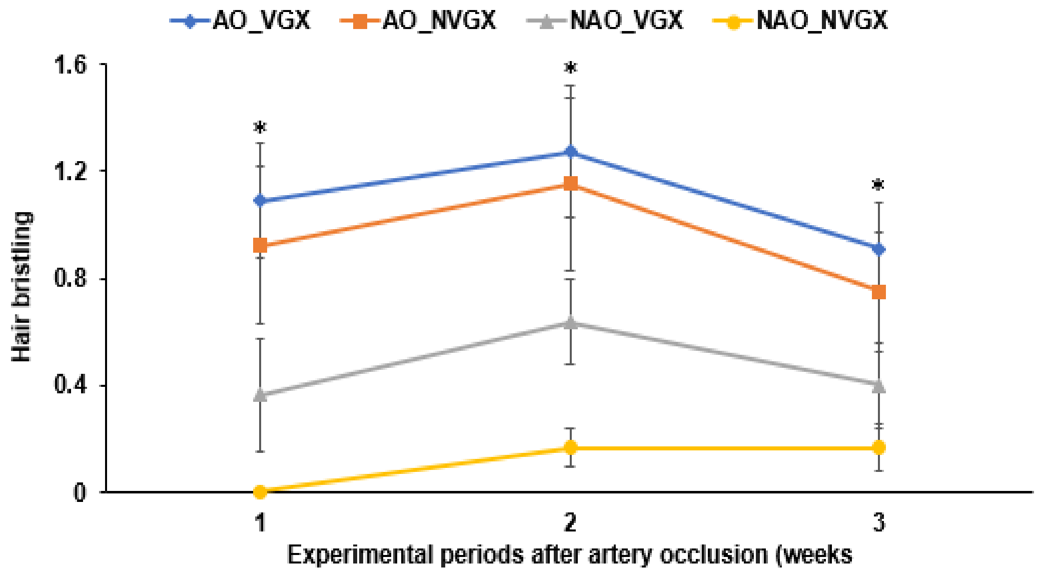

C. Reflexes

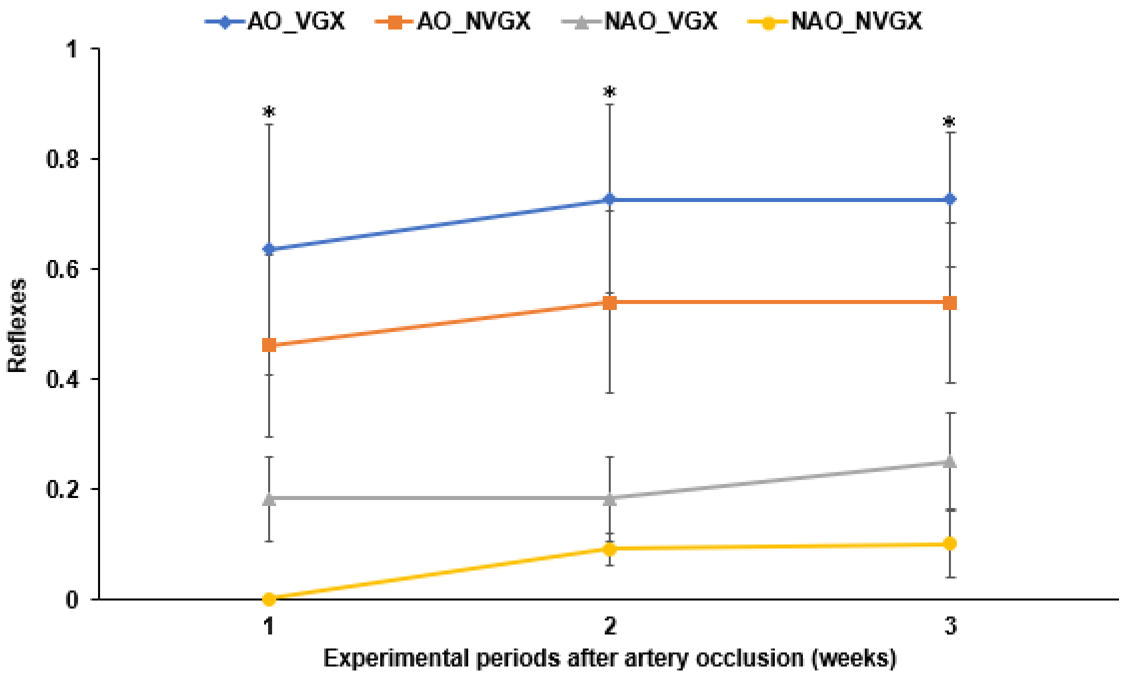

D. Posture

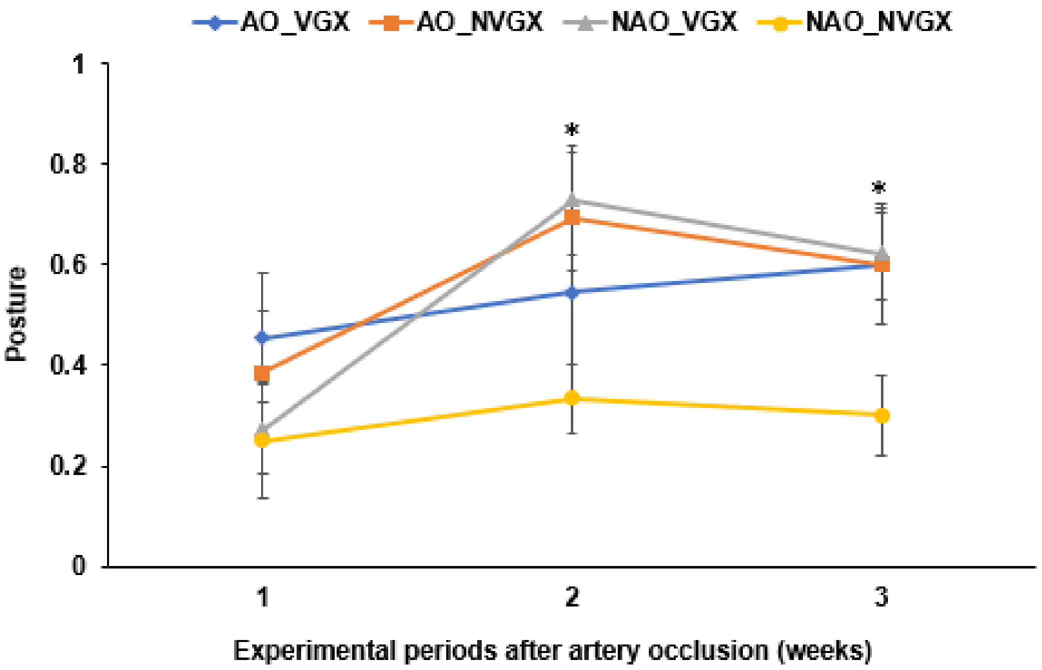

E. Walking pattern

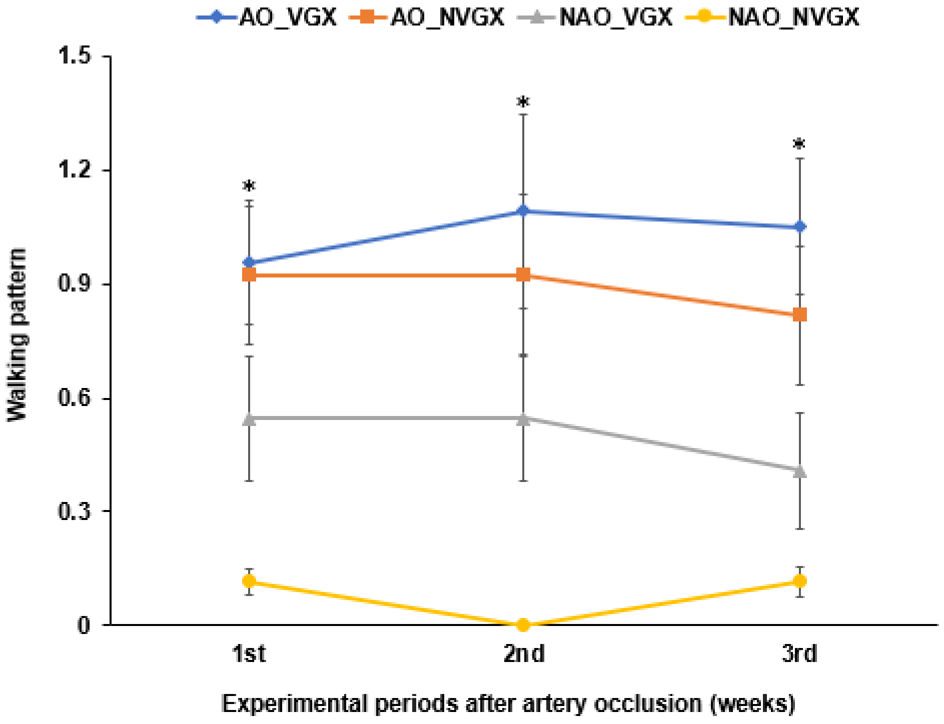

Supplement: Supplementary file 1 [file ijms-25-07831-s001.zip › supplementary figure.pdf]
