# Supplementary material for: Vagus Nerve Suppression in Ischemic Stroke by Carotid Artery Occlusion: Implications for Metabolic Regulation, Cognitive Function, and Gut Microbiome in a Gerbil Model
Source: Int J Mol Sci. 2024 Jul 17;25(14):7831. doi: 10.3390/ijms25147831 (PMC11276658; doi:10.3390/ijms25147831)
Supplement: Supplementary file 1 [file ijms-25-07831-s001.zip › supplementary table.pdf]

Table S1. A high-fat diet nutrient composition.

Nutrient composition	Amount (g)	Energy percent (En%)
Casein	197	17.0
Methionine	3	
Corn starch	266	39.9
Sucrose	200	
Cellulose	34	
Corn oil	20	43.1
Lard	205	
Mineral	35	
Vitamin	10	
Choline	2	
Cholesterol	25	

Energy percent of each macronutrient in a high-fat diet was calculated by dividing energy from each macronutrient amount by total energy of diet.
